# Primate dental function and evolution: longitudinal 3D tooth wear in wild baboons

**DOI:** 10.1017/ehs.2026.10041

**Published:** 2026-03-24

**Authors:** Ian Towle, Luca Fiorenza, Kristin L. Krueger, Clifford J. Jolly, Jane Phillips-Conroy

**Affiliations:** 1 Biomedicine Discovery Institute, Department of Anatomy and Developmental Biology, Monash University, Melbourne, VIC, Australia; 2 Department of Anthropology, Loyola University Chicago, Chicago, IL, USA; 3 Department of Anthropology, New York University, New York, NY, USA; 4 Department of Neuroscience, Washington University School of Medicine in St. Louis, St. Louis, MO, USA

**Keywords:** tooth wear, *Papio*, *WearCompare*, tooth attrition, hybridization

## Abstract

Tooth wear constrains feeding efficiency, life history, and survival in mammals, yet its progression in wild populations remains poorly understood. We use high-resolution 3D analysis to quantify occlusal tissue loss over a 3-year period in the upper premolars and molars (P3-M3; *n* = 70) of wild baboons (*Papio*). Our sample includes olive baboons (*P. anubis*) and naturally occurring olive-hamadryas hybrids (*P. anubis* × *P. hamadryas*) from Awash National Park, Ethiopia. We calculate mean values for tooth types, visualize tissue loss across occlusal surfaces, and compare individuals by age, sex, and hybrid status. Molars lost tissue faster than premolars (molars: 0.13 mm^3^/mm^2^/year; premolars: 0.08 mm^3^/mm^2^/year), with the bulk of wear shifting from lingual to buccal cusps in older individuals. The rate of tissue loss did not increase with age, despite greater dentine exposure. There was no clear difference in wear patterns relating to sex or hybrid status, although subtle sex-related differences in P3 wear patterns were observed. These findings demonstrate the adaptive significance of gradual tissue loss in preserving dental function and establish comparative baselines for interpreting wear patterns in extinct primates, where dental remains often provide the primary record of diet and behaviour.

## Graphical abstract

**Figure:**
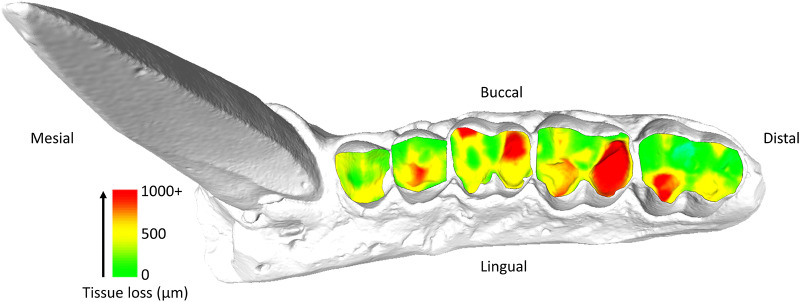

## Social media summary

Adaptive tooth wear in wild baboons highlights patterns preserving dental function over time.

## Introduction

Tooth wear is a central aspect of mammalian biology and physiology, as teeth come into direct contact with dietary items and the environment throughout an individual’s lifetime. In many taxa, including primates, the progression of tooth wear affects not only an individual’s feeding efficiency but also its reproductive success and, ultimately, lifespan (Ungar, 2015). Consequently, diverse mechanisms have evolved that maintain dental function, and primate teeth undergo predictable morphological changes that help prolong functional efficiency (e.g., Kay, 1975; Knight-Sadler and Fiorenza, 2017). Understanding these dynamic processes is therefore crucial for interpreting the interaction between the dentition and ecological pressures. Moreover, these predictable and phasic changes in dental morphology can provide additional data for phylogenetic studies, complementing analyses based solely on the morphology of unworn molars (Ungar & M’Kirera, 2003).

In macrowear analyses, researchers commonly use grade scales based on enamel wear facets and the extent of dentine exposure (e.g., Scott, 1979; Smith, 1984). Other studies quantify wear as a continuous variable by measuring the percentage of the occlusal surface that consists of exposed dentine (e.g., Galbany et al., 2014; Pampush et al., 2018). For practical reasons, studies of tooth wear, both macro and micro, typically assess wear at a single-time-point per individual. Such cross-sectional data have been instrumental in behavioural reconstructions but cannot fully capture the trajectory of tooth surface change over an individual’s lifetime. Longitudinal studies of tooth wear in wild primates are in contrast rare (Froehlich et al., 1981; King et al., 2005; Phillips-Conroy et al., 2000; Teaford & Glander, 1996). This is understandable, as it involves repeatedly locating, capturing, and safely immobilizing the same individuals, followed by obtaining moulds or scans under field conditions.

Wear does not necessarily progress evenly, and neither dentine exposure nor enamel facet development always reflects the incremental or phasic nature of dental tissue loss. A major contributor is the heterogeneity of dental tissue properties, not only the substantial difference in mechanical properties between enamel and dentine but also variation within each tissue (e.g., from inner to outer layers; Andrejovská et al., 2023; Foster, 2022; Towle et al., 2023a). Because dentine is considerably softer, it has long been assumed that tissue loss should accelerate once the enamel cap is breached. However, more subtle temporal variation is also likely. As overall tooth morphology changes with wear, including as cusp height is reduced, occlusal dynamics may shift (Lucas, 2004). For example, if certain cusps function as ‘guides’ that influence how opposing surfaces meet during mastication, the loss of these structures may alter the masticatory cycle and presumably modify wear progression (Ungar et al., 2018). In addition, changes in which teeth are in occlusion at different life stages can reshape the masticatory cycle, occlusal relationships, bite forces, and the way food interacts with specific tooth surfaces (Molnar & Ward, 1977; Schwartz, 2000).

Commonly used methods for assessing tooth wear may be limited in their ability to capture such fine-scale variation in wear across a tooth’s lifespan. Grade-based scoring systems (e.g., Scott, 1979; Smith, 1984) generate ordinal data that inherently group disparate stages of wear, making it challenging to align these categories with the actual progression of tissue loss. Some grade transitions may encompass substantial tissue removal, whereas others may reflect very little change. Quantitative measures, such as per cent dentine exposure (PDE), similarly provide an incomplete picture because they do not capture total tissue loss. Considerable enamel may be removed before dentine is first exposed, and later in the functional life of a tooth, when dentine dominates the occlusal surface, PDE may change only minimally despite continued substantial tissue loss (Krueger et al., 2025). These limitations underscore the need for longitudinal assessments of tissue loss, both to evaluate how wear progresses over time and to develop approaches that can complement or improve existing single-time-point methods.

Using methods not previously applied to wild primates, in this study we document loss of dental tissue at the occlusal surface of molars and premolars in individual baboons (*Papio*). The subjects were olive baboons (*P. anubis*) and natural hybrids between olive and hamadryas (*P. hamadryas*) baboons, inhabiting the Awash National Park, Ethiopia. Animals were captured, tooth-cast, and released on two occasions, enabling quantification of dental tissue loss across occlusal surfaces over a 3-year interval. These data were then used to compare patterns of attrition to assess how these methods perform in wild primate populations, and provide initial comparisons across age, sex, and hybridization status. Like other *Papio* populations (Altmann, 1998; Barton & Whiten, 1994), the Awash baboons consume a diverse, primarily vegetarian diet that varies seasonally. Despite this diversity, the majority of their diet comes from a limited number of plant species. Seasonal shifts are reflected in their consumption of different plant parts, including leaves (from grasses, shrubs, and trees), fruits, flowers, grass shoots, seeds, sedge corms, and flowers (Nystrom, 1992; Nystrom et al., 2004; Phillips-Conroy et al., 2000).

We predict that tooth wear will vary by tooth type and among individuals (Galbany et al., 2020; Morse et al., 2013; Teaford, 1983), progressing slowly in younger animals until enamel is breached, after which rates of tissue loss will accelerate (Galbany et al., 2011a; Phillips-Conroy et al., 2000). We further expect males to exhibit greater absolute tissue loss owing to their larger body size and associated dietary demands, specifically, increased nutritional requirements in larger males may result in longer chewing times and, consequently, greater wear. However, after controlling for age and tooth size, we anticipate broadly similar wear patterns between the sexes, consistent with previous findings for dentine exposure in wild baboon populations (Galbany et al., 2011a). Nevertheless, earlier studies of free-ranging baboons (Bramblett, 1967), as well as more recent work on captive populations, have reported substantially greater wear in males than females that cannot be explained by differences in age or tooth size (Krueger et al., 2025; Towle et al., 2024). Comparable sex-related differences in wear patterns have also been documented and explored in other Papionini primates (e.g., Guatelli‐Steinberg et al., 2022; Nass, 1981). More broadly, studies of other primates have frequently failed to detect expected sex differences in tooth wear, including in highly sexually dimorphic taxa such as extant great apes (Fiorenza et al., 2024; Harty et al., 2022). Potential sex differences therefore warrant explicit investigation.

We also predict potential group-level differences in wear patterns linked to hybridization (Alberts & Altmann, 1995; Jolly, 2001; Swedell & Plummer, 2012). In particular, previous work on captive baboons with documented hybrid ancestry has shown dental changes with clear functional implications, including a higher frequency of supernumerary teeth, reduced tooth size in some elements, and other abnormalities such as rotation and crowding (Ackermann et al., 2014), all of which could influence occlusion and masticatory dynamics.

The overall aim of this exploratory study (*n* = 70 teeth from 21 individuals) is to evaluate the feasibility of these methods and to identify potential patterns worthy of further investigation using these new techniques. We first evaluate the application of these techniques to wild primates by examining scan alignment relative to comparable time points in captive baboons and human samples (O’Toole et al., 2019, 2020; Towle et al., 2024; Towle and Fiorenza, 2025). We then examine how heat-map patterns of tissue loss and wear severity vary among groups, between males and females, and across age classes. Finally, to contextualize this approach, we compare our longitudinal wear data with two established single-time-point methods, PDE and the Krueger–Scott method (KSM; Krueger et al., 2025).

## Methods

Our study sample comes from three baboon troops (H, D, and G) living in and around the hybrid zone of Ethiopia’s Awash National Park. We present a longitudinal 3D analysis of tooth wear progression in these wild baboons (*P. hamadryas* × *P. anubis*). *Papio* is a widespread genus with a complex evolutionary history, including both recent and ancient hybridization events that complicate interpretations (Sørensen et al., 2023). Extensive research has been carried out across fieldwork, paleontological research, and molecular contexts (e.g., Gilbert et al., 2018; Jolly & Brett, 1973; Sørensen et al., 2023). Following the current near-consensus (e.g., Sørensen et al., 2023), we classify *P. hamadryas* and *P. anubis* as full species.

In one study group (group H; see Phillips‐Conroy & Jolly, 1986 for group assignment, group range, and details), most individuals show genetic and phenotypic evidence of multi-generational admixture. Group H has been considered to lie at the phenotypic centre of the hybrid zone (Woolley-Barker, 1999; Bergman et al., 2008). In contrast, baboons from adjacent groups (D and G), consist of individuals that display more purely olive baboon morphology and behaviour (Nystrom et al., 2004; Phillips‐Conroy & Jolly, 1986; Phillips‐Conroy et al., 1991). Here, we refer to the former as ‘hybrids’ and the latter as ‘olive’.

Impressions of the upper left dentition were taken from individuals in Groups D, G, and H in both 1995 and 1998 (*n* = 70 teeth from 21 individuals; Table 1), allowing this assessment of tooth wear progression over a 3-year period. All dental moulds were taken in July or early August of each year (1995 and 1998), with the time between impressions therefore varying minimally, and by no more than 2 weeks from a 3-year period (average of 5 days either side of exactly 3 years; Table 1).

**Table 1.** Summary of sample studied

Individual	Teeth	Sex	Taxon	Group	Age class	First mould	Second mould
11007	P3, P4, M1, M2	Female	Hybrid	H	3	3/07/1995	17/07/1998
11009	P3, P4, M1, M2	Male	Hybrid	H	3	3/07/1995	9/07/1998
11011	P3, P4, M1, M2	Male	Hybrid	H	2	3/07/1995	4/07/1998
11022	P3, P4, M1, M2, M3	Female	Hybrid	H	4	5/07/1995	9/07/1998
11023	P4, M1, M2, M3	Female	Hybrid	H	4	5/07/1995	12/07/1998
11018	P3, P4, M1, M2	Male	Hybrid	H	4	5/07/1995	4/07/1998
11019	P3, P4, M1, M2	Male	Hybrid	H	3	5/07/1995	4/07/1998
11015	M1	Male	Hybrid	H	1	3/07/1995	4/07/1998
11020	M1, M2	Male	Hybrid	H	2	5/07/1995	4/07/1998
11030	P3, P4, M1, M2, M3	Male	Hybrid	H	4	7/07/1995	6/07/1998
11038	P3, P4, M1, M2, M3	Male	Hybrid	H	4	7/07/1995	6/07/1998
11039	P3, P4, M1, M2, M3	Female	Hybrid	H	3	7/07/1995	10/07/1998
11021	M1	Male	Hybrid	H	1	5/07/1995	4/07/1998
11029	M1, M2, M3	Male	Hybrid	H	4	7/07/1995	4/07/1998
11006	P3, P4, M1, M2, M3	Male	Hybrid	H	4	3/07/1995	9/07/1998
11026	M1	Female	Hybrid	H	1	5/07/1995	4/07/1998
10862	P3, P4, M1, M2	Female	Olive	G	2	19/07/1995	10/07/1998
11050	M1	Male	Olive	G	1	19/07/1995	5/07/1998
11053	P3, P4, M1, M2	Male	Olive	D	2	22/07/1995	5/08/1998
11060	M1	Female	Olive	D	1	23/07/1995	1/08/1998
10947	P3, P4, M1	Male	Olive	D	2	22/07/1995	5/08/1998

*Note*: Each row is an individual, with the second column listing teeth for each individual.

All individuals are wild baboons (*Papio*) from within the Awash National Park (Ethiopia) hybrid zone (*P. hamadryas* × *P. anubis*). Hybrid refers to the group (H), whereas the other two groups are more uniformly Olive-like. Lastly, the date of moulding for each individual in the 2 separate years (1995 and 1998) is presented.

The procedure for dental moulding has been described in detail elsewhere (Phillips‐Conroy et al., 1991) and is summarized here. Each baboon was individually captured in a drop-door box trap, sedated with ketamine, and its upper left dentition was brushed and rinsed with water. A dental impression was then obtained using a water-based alginate impression material (Jeltrate Fast Set; Dentsply Caulk, Milford, DE, USA), placed in a prepared tray. The filled tray was positioned over the occlusal surface and held firmly in place for 1–2 min. Immediately after the mould was removed from the teeth, it was filled with dental stone slurry (Castone dental Stone; Ransom & Randolph, Maumee, OH, USA), and agitated to dislodge air bubbles. The dental stone cast was allowed to set hard, then removed and labelled.

Casts were digitized using photogrammetry techniques. Each cast was scanned on a rotary table with a structured-light 3D scanner (HDI Advance R3X13; software: FlexScan3D; minimum point-to-point distance: 0.065 mm; Moore & Kettler, 2018). Calibration was regularly performed using a calibration board, and a sub-sample was cross-validated using direct caliper measurements to ensure scanning accuracy (Moore & Kettler, 2018). Each tooth was segmented from the full cast to create individual STL files, and any minor artefacts were removed using Meshlab and MeshMixer (Cignoni et al., 2008).

### Wear measurements

The two 3D meshes representing a particular tooth (i.e., one from 1995 and one from 1998) were superimposed in *WearCompare* (Leeds, UK: https://leedsdigitaldentistry.com/wearcompare/) to estimate tissue loss (O’Toole et al., 2020, 2019; Towle et al., 2024). The superimposition process involved aligning the surfaces using the global recognition feature with an iterative closest point algorithm, followed by selective surface alignment focusing on buccal and lingual lateral enamel (O’Toole et al., 2020; Towle et al., 2024). Alignment quality was assessed based on comparisons with O’Toole et al. (2020), in which poor alignment was defined as less than 75% of data points coinciding within 25 µm of each other. All *WearCompare* analyses were performed by a single operator (IT).

Following O’Toole et al. (2020), loss of dental tissue volume was quantified using the occlusal surface as the measurement area. The main variables recorded for each tooth were total volume loss (mm^3^) and volume loss per mm^2^ of surface area. Additional metrics included maximum surface deviation, which is the point at which the most tissue has been removed (measured vertically from the occlusal surface in microns), and the mean surface deviation lost across the entire occlusal surface. Broad comparisons are assessed visually from the resulting heat maps, including which side of the occlusal surface is most affected, and any evidence for atypical tissue loss such as fractures or malocclusion.

We also compared the tissue loss results with two other wear assessment methods commonly used in primatology and bioarcheology/paleoanthropology: (1) A modified Scott scoring system based on wear facets and dentine exposure (KSM; Krueger et al., 2025; Scott, 1979), adapted to accommodate cercopithecid bilophodont molars (following Krueger et al., 2025), recorded using the 3D digital models; (2) PDE across the occlusal surface was quantified following Galbany et al. (2011a, 2014), using 2D screenshots of the 3D models in place of digital camera images. Measurements were taken in Fiji (Schindelin et al., 2012) by using the segmented line/ROI tool to delineate areas of dentine exposure (combining all islands when present). Total occlusal area was then recorded using the same approach, and PDE was calculated as (dentine exposure/total occlusal area) × 100.

A Spearman’s rank correlation was undertaken to assess relationships between the continuous numerical variables (PDE and tissue loss data) and the ordinal categorical variable (KSM). To assess whether changes in dentine exposure (PDE) corresponded with volumetric tissue loss (i.e., two continuous variables) a Pearson correlation was also conducted between the difference in PDE values from 1995 to 1998 and tissue loss data. Only teeth with available data for both years were included. Given the ordinal nature of KSM, a Kruskal–Wallis test was used to determine if significant differences in tissue loss were present across different score categories. All statistical analyses (with the significance level set at α = 0.05) were conducted in R (version 4.2.2; R Core Team, 2019).

### Age, sex, and taxon comparisons

To examine potential effects of sex, taxon, and age class on tooth wear, volume loss per mm^2^ (mm^3^/mm^2^) was compared across four age categories. Age classification was based on dental eruption, with the final category (4) additionally defined by third molar dentine exposure: (1) first molar in occlusion; (2) first and second molars in occlusion; (3) first, second, and third molars in occlusion; and (4) all molars in occlusion plus visible dentine exposure on the third molars. Given the small sample size and uneven representation across groups (e.g., sex and hybrid status within each age class), this component of the study emphasizes descriptive analyses of tissue loss and comparisons of overall wear patterns derived from the resulting heat maps. Figures (scatterplots, regression plots, and boxplots) were generated using Python’s SciPy and Seaborn libraries. The data supporting the findings of this study are openly available in the Supplemental File accompanying this article.

## Results

### Validity of methods and tissue loss averages

**Figure 1.**
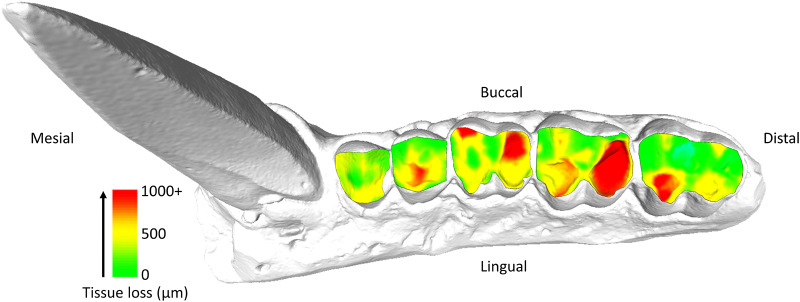
Specimen 11038, visualization of tissue loss across the occlusal surface of the maxillary premolars and molars (P3-M3). Each tooth was individually measured for tissue loss (see Methods) before being reassembled on the original model for this figure.

**Figure 2.**
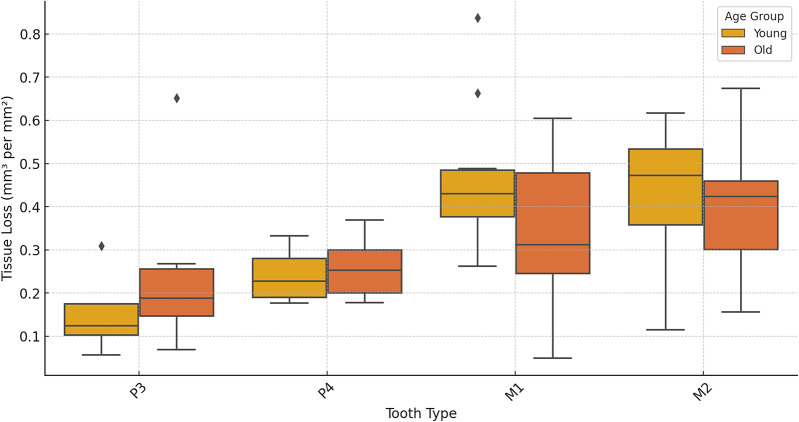
Tissue loss (mm^3^/mm^2^) for each tooth split into young (age class 1 and 2) and older (age class 3 and 4) individuals for each. Third molars are not included due to no samples in the young age class.

Premolars showed an average tissue loss of 2.44 mm^3^/year (0.08 mm^3^/mm^2^), while molars lost an average of 8.38 mm^3^/year (0.13 mm^3^/mm^2^). In the molars, the highest levels of loss were found in lingual cusps, though buccal cusps wear increased progressively with age class (Fig. 1). Overall occlusal tissue loss was broadly similar across age classes for all tooth types (Fig. 2). No clear differences were observed in occlusal wear patterns or total tissue loss between hybrid and olive baboons (Table 2). Alignment accuracy was high, with >75% of alignment points on buccal and lingual lateral enamel between the 1995 and 1998 3D models falling within 25 μm of each other, with an overall average of 87.39%. This is similar to, or even better, than comparable alignment for captive baboons and different human samples (O’Toole et al., 2019, 2020; Towle et al., 2024; Towle and Fiorenza, 2025). This confirms the reliability of the alignment procedure for assessing occlusal tissue loss in this wild primate population (Fig. 1).

**Table 2.** Tissue loss averages for different teeth and categories, for both total tissue loss and with tooth size taken into account (mm^3^/mm^2^), along with the number of samples and standard deviation

Category	*N*	Volume loss (mm^3^)	Volume loss (mm^3^/mm^2^)
Premolars (P3/P4)	27	7.33 ± 4.36	0.23 ± 0.12
Molars (M1/M2/M3)	43	25.14 ± 11.88	0.39 ± 0.16
Hybrid baboons	57	18.43 ± 13.18	0.32 ± 0.15
Olive baboons	13	17.56 ± 12.73	0.37 ± 0.23
Males (all teeth)	46	19.27 ± 13.58	0.34 ± 0.18
Females (all teeth)	24	16.35 ± 11.90	0.30 ± 0.14

### Tissue loss pattern and variation

**Figure 3.**
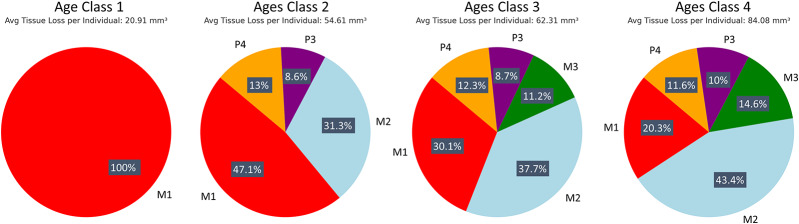
Progression of tissue loss across age classes. Tissue loss data are shown for each age class, highlighting the increasing involvement of the second and third molars through time.

Patterns of tissue loss changed over time, reflecting the shifts in the role of each tooth in mastication (Fig. 3). In younger individuals, the first molar contributed the most to overall tissue loss, but by age class 4, its contribution declined to a quarter of total posterior tooth tissue loss. The second and then third molars showed increasing proportions of tissue loss with age class (Fig. 3). Although males exhibited higher absolute volume loss (mm^3^), values were very similar between sexes once tooth size was taken into account (mm^3^/mm^2^). Ranges for each tooth type overlapped extensively, with males showing slightly higher mean values for all teeth except the third premolar, for which females showed higher averages (Supplementary Figure S1).

**Figure 4.**
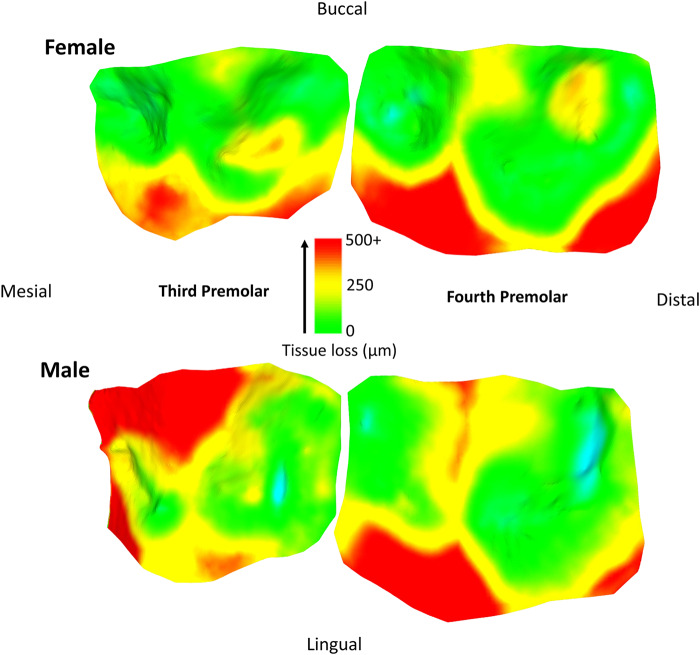
Specimen 10862 (female) and 11019 (male). Note the continuations of wear from the third premolar to the fourth premolar, with similar wear in male and female on the fourth premolar, but different wear for the third premolar, potentially relating to the proximity and involvement of the canine honing complex. Scale bar in microns. Each tooth was processed individually for tissue loss, and put back together for these figures. Areas of blue coloration are erroneous ‘increase’ in tissue, relating to a small bit of food/saliva that was not removed by the brushing, slight artefacts on the casts or slight errors in alignment of the two meshes.

**Figure 5.**
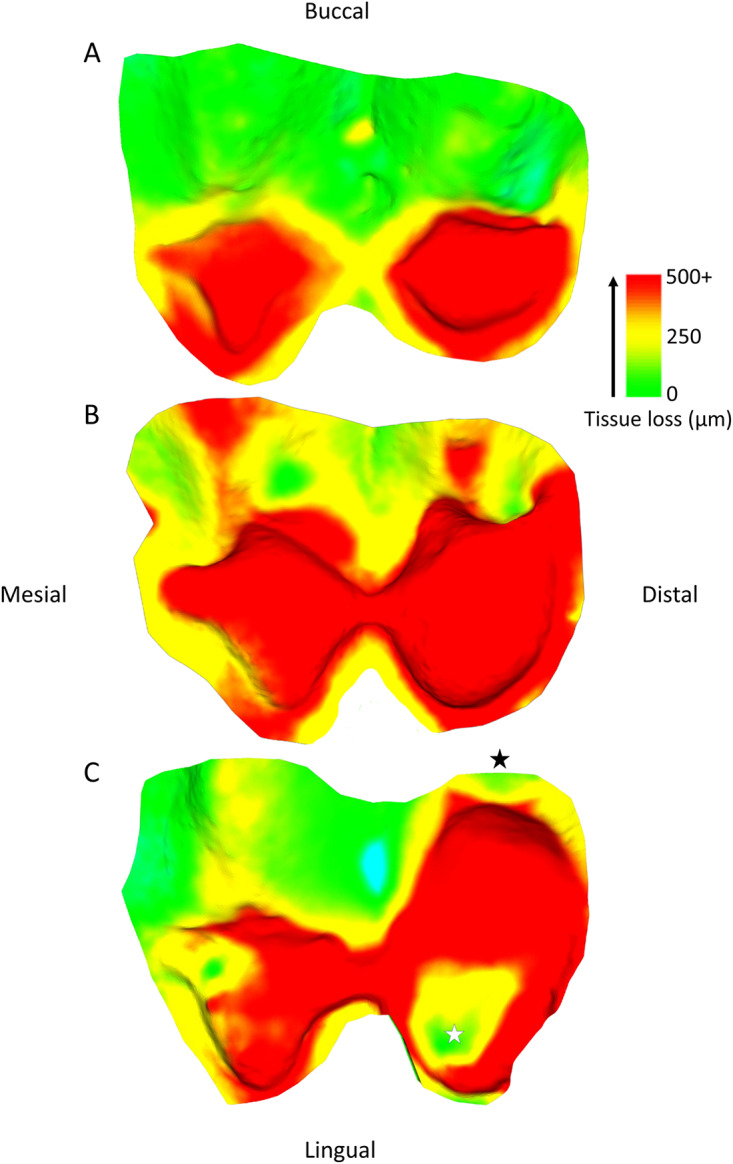
Changing wear pattern across the occlusal surface with age, using three individuals at different stages of second molar wear. (A) specimen 11022; (B) specimen 11023; (C) specimen 11006. A tooth fracture is highlighted by the black star, and an area of little volume loss within the exposed dentine is marked by the white star, likely associated with tertiary dentine formation. The blue area in (C) is a small artefact. Scale bar in microns.

Tissue loss patterns across the occlusal surface were consistent among individuals. In younger baboons, the highest levels of tissue loss occurred on cusp tips, especially the lingual cusps, with notable exceptions found in male third premolars (Fig. 4). This variation in premolar wear between males and females may reflect differences related to the sexually dimorphic canine-honing complex. Although the P3 itself is not a direct component of the honing complex, its role in mastication is likely slightly different between the sexes by its proximity to this sexually dimorphic feature (Galbany et al., 2011b). However, further research is needed to test this hypothesis. Overall, premolars seem to display a more stable rate of tissue loss than molars, with less variability within and across age cohorts (Figs. 2 and 3). In molars, tissue loss is initially concentrated on the lingual cusps, but later becomes more evenly distributed across the occlusal surface, with buccal cusps eventually showing the most tissue loss in older individuals (Fig. 5; see also first molar in Fig. 1). The formation of tertiary dentine, initially in lingual cusps, likely plays a crucial role in maintaining tooth function and may help explain why tissue loss does not accelerate once dentine exposure begins (Fig. 5). Other types of tissue loss, such as tooth fractures, were also detected in the sample (Fig. 5).

### Comparing tissue loss analysis with conventional wear scoring methods

Spearman correlation analysis shows a very weak correlation (Spearman’s *ρ: ρ* = 0.001) between change in PDE and tissue loss (mm^3^/mm^2^), indicating no meaningful relationship between these measures (Supplementary Figure S2). This was supported by a Pearson correlation test with the change in PDE from 1995 to 1998 showing a weak, non-significant, correlation with tissue loss (Pearson correlation test: mm^3^/mm^2^; *r* = 0.11, *p* = 0.49). The correlation between changes in KSM and tissue loss was stronger, but still weak (Spearman’s *ρ: ρ* = 0.175), suggesting that KSM is a slightly better predictor of tissue loss than PDE, though neither method provided a strong predictor of tissue loss. However, age showed a strong positive correlation with changes in PDE (Spearman’s *ρ: ρ* = 0.599), while neither KSM (Spearman’s *ρ: ρ* = 0.085) nor tissue loss (Spearman’s *ρ: ρ* = − 0.172) showed a clear relationship with age. Kruskal–Wallis tests confirmed a significant effect of age class on PDE (Kruskal–Wallis tests: *H* = 16.07, *p* = 0.001), whereas KSM (Kruskal–Wallis test: *H* = 2.28, *p* = 0.516) and tissue loss (Kruskal–Wallis test: *H* = 2.96, *p* = 0.398) did not significantly differ across age classes. These results indicate that PDE accelerates with age (i.e., the rate of wear increases with age), while KSM change and tissue loss do not (Supplementary Figure S3).

## Discussion

This study found annual tissue loss values of 0.08 mm^3^/mm^2^ for premolars and 0.13 mm^3^/mm^2^ for molars. These serve as comparative baselines for upper posterior teeth within Papionini. Our findings are consistent with established patterns in *Papio* and other cercopithecids, rapid post-eruption wear at the cusp tips followed by predictable occlusal progression (Bramblett, 1969; Phillips-Conroy et al., 2000). As in earlier work, tissue loss initially concentrates on the lingual surfaces of upper molars, where occlusal forces from mastication preserve sharp shearing edges despite substantial attrition (e.g., Kay, 1977; Towle et al., 2021). These results lend further support to functional interpretations of occlusal dynamics and highlight the value of high-resolution wear tracking in refining such models.

Contrary to expectations, loss rates did not increase with age or following dentine exposure. Instead, we observed a consistent pattern across individuals, in which the bulk of wear initiates on lingual cusps and then transitions to buccal cusps after a threshold of dentine exposure is reached, without a corresponding rise in total tissue loss. When adjusted for tooth size, there were no obvious differences in wear severity or occlusal patterns between sexes, supporting prior research on other wild baboon populations (Galbany et al., 2011a). The possible exception was the P3 with evidence for an influence of sexual dimorphism in the canine-honing complex (Delezene, 2015; Jolly, 2001).

Yet, more broadly, individual differences in behaviour, dietary preferences, and access to food resources, often shaped by social rank, age, and sex, have been shown to influence tooth wear (Kilgore, 1989; Post et al., 1980). Prior research has also documented subtle variations in social behaviour and diet within the Awash hybrid zone (Bergman et al., 2008; Nystrom et al., 2004; Phillips‐Conroy et al., 1991). Research on wild mantled howler monkeys also suggests that life events such as pregnancy and lactation can significantly impact tooth wear (Teaford & Glander, 1996). Despite this, the present study and those on other living primate taxa with substantial body sexual dimorphism have failed to detect obvious sex differences in tooth wear patterns and progression (Fiorenza et al., 2024; Harty et al., 2022). Further investigation is therefore needed to determine how sexual dimorphism, including life history factors, influences variation within specific age and sex categories, particularly through the integration of direct field observations.

We also did not find any obvious differences in overall tissue loss or occlusal wear patterns across hybrid and olive groups. Previous studies on *Alouatta* (howler monkeys) found conserved wear patterns across microhabitats (Dennis et al., 2004), while microwear studies on these same baboons similarly indicated minimal environmental or hybridization impact (Nystrom et al., 2004). The present study does not offer any support that hybridization status in itself impacts tooth wear progression in baboons, but larger samples are required to test this further. Furthermore, there was also no indication of malocclusion or pathology in wear patterns in these hybrid baboons, and therefore no indication of reduced fitness compared to the olive baboon samples, at least in terms of tooth wear and associated pathologies.

All *Papio* taxa have been observed consuming items that involve digging in soil or collecting foods with grit adhering to their surfaces. Although baboons often attempt to remove grit before eating, substantial amounts are inevitably ingested (Galbany et al., 2011a). Given that fine, wind-borne dust often accumulates on vegetation eaten by the baboons, complete removal would be extremely challenging (Nystrom et al., 2004). Similar behaviour is seen in other Papionini species, such as *Macaca fuscata*, which try to wash grit or sand from their food items, yet consume significant amounts (Sarabian & MacIntosh, 2015), leading to considerable dental tissue loss (Towle et al., 2022). These observations underscore the importance of environmental and ecological factors in shaping tooth wear, particularly in the context of Anthropocene-driven changes to wild primate habitats. Ongoing research further suggests that the incorporation of grit into the oral cavity during mastication is more complex than previously assumed, with multiple environmental and behavioural factors influencing its entry and distribution (Fannin et al., 2021; Geissler et al., 2018; Spradley et al., 2016; Ungar et al., 1995). Applying 3D wear-analysis techniques in comparative studies of baboons across a wide range of habitats may therefore provide valuable insights into how changing environments alter tooth wear patterns.

The hardness of environmental minerals like quartz can contribute to continual tissue loss through gradual abrasion, but, in some cases, also larger-scale occlusal edge fractures (Fannin et al., 2021; Geissler et al., 2018; Towle et al., 2017; Ungar et al., 1995). The contribution of different types of wear to the accumulation of tissue loss in the Awash National Park baboons has been discussed previously, especially regarding abrasion and tooth fractures. The latter is commonly observed in these, and other, baboons (Nystrom et al., 2004; Phillips-Conroy et al., 2000; Towle & Loch, 2021). Due to the focus of the present study on tissue loss across whole occlusal surfaces, distinguishing the individual contributions of small fractures (e.g., edge chipping) versus the more gradual abrasion and attrition remains challenging. However, tooth fractures appear to play a crucial role in tissue loss, especially in non-functional cusps. Moreover, fractures seem to influence localized wear patterns both before and after their occurrence, underscoring the complex interactions between different types of wear over time (e.g., Fig. 5c; Towle et al., 2024 Fig. 6).

**Figure 6.**
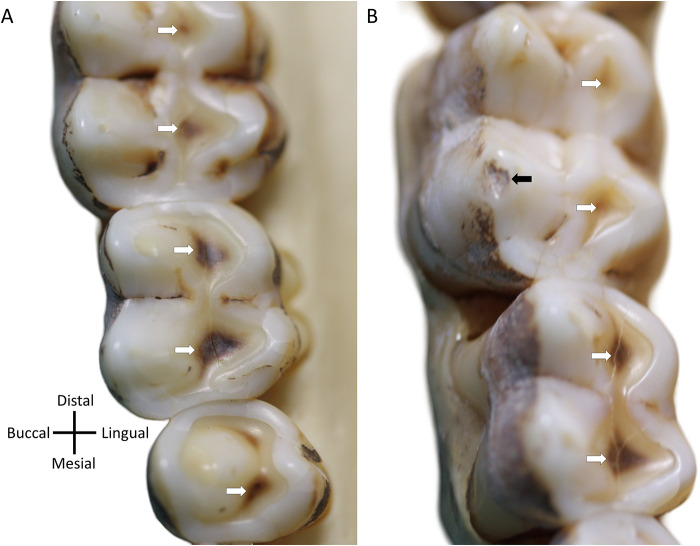
Two wild female Hamadryas baboon (*Papio hamadryas*), curated at the Primate Research Institute (PRI), Japan, showing tertiary dentine formation in response to tissue loss (white arrows), likely as part of a normal physiological processes for optimizing tooth function through time. (A) PRI 5785, upper left posterior teeth; (B) PRI 5794, upper left posterior teeth (black arrows highlight an antemortem chip). *Note*: These two individuals are not part of the tooth wear analysis in the present study and are just to highlight how *P. hamadryas* show extensive evidence of developing tertiary dentine early after dentine is exposed through wear. Second molars at top in both cases.

Our findings align with those of the Amboseli Baboon Research Project on wild yellow baboons (*Papio cynocephalus*), which found that dentine exposure accelerates with age (Galbany et al., 2011a), as well as an earlier study on Ethiopian and Tanzanian baboons (Phillips-Conroy et al., 2000). However, we did not observe an age-related change in tissue loss, at least in the hybrid sample for which more data were available. This discrepancy is likely a function of tooth morphology, extensive tissue loss early in a tooth’s functional life may result in only small dentine patches on cusp tips, whereas similar tissue loss later in life can uncover disproportionately larger areas of dentine. As wear becomes severe, dentine exposure again slows because the remaining enamel is confined to the tooth’s lateral walls. This effect may be more striking in certain taxa and highlights the potential limitations in dentine exposure as a wear metric, particularly in comparing species that include groups with very thick enamel (Martin et al., 2003; Morse et al., 2023).

The KSM (Krueger et al., 2025) appears to track overall tissue loss progression more accurately than PDE, as both tissue loss and KSM remained approximately the same across the four age classes. This suggests that KSM may better capture the progression of tooth wear, likely due to subtler variation especially in early stages of wear (i.e., before dentine is exposed using enamel wear facet development). Consequently, both PDE (which allows for quantitative analysis) and KSM (which enables faster data collection and may better represent actual tissue loss through time) have distinct advantages. A practical approach would be to use detailed tissue loss analyses on a smaller subsample (i.e., using two time points) and employ single-time-point data (PDE or KSM; or other methods, e.g., see Pampush et al., 2024) on a larger sample, with the smaller, more detailed analysis serving to calibrate the broader dataset. This approach may also benefit paleontological research by using an extant close relative as an analogue for the tissue loss subsample.

Why does tissue loss not accelerate as PDE increases, given dentine is much softer than enamel (Towle et al., 2023b)? One possible explanation is that, as later erupting teeth emerge, the total occlusal surface area increases, distributing wear across a greater number of teeth. Additionally, more anterior and already worn molars may drop slightly below the occlusal plane, reducing direct contact with opposing teeth and their exposure to occlusal forces (Jolly, 1972). Age-related changes in the dentition and mastication may further alter how loads are distributed, with forces shifting across different teeth and tooth positions as teeth erupt and individuals’ age (Galbany et al., 2011b). Another important factor is the structural complexity of dental tissues. A significant and often overlooked contributor to moderating tissue loss is the formation of new dentine after tooth eruption. Tertiary dentine appears to play crucial roles in maintaining the functionality of baboon posterior teeth (Fig. 6). The consistent wear patterns observed in our sample suggest that this new dentine formation is vital in preserving tooth function. For example, as the lingual cusps wear down, tertiary dentine is quickly deposited, helping to preserve the shearing edge and protect the pulp. Over time, wear shifts to the buccal cusps, where tertiary dentine formation is also evident (Fig. 6). Recent research suggests that some primate taxa may have evolved the ability to produce tertiary dentine more efficiently and at faster rates and be closely linked to other tooth morphological and structural features (Selig et al., 2021; Towle, 2019).

Many factors shape or constrain the size and form of individual teeth within a dentition (e.g., Cano‐Fernández & Gómez‐Robles, 2021; Carter & Worthington, 2016; Evans et al., 2016; Machado et al., 2023; Roseman & Delezene, 2019). To address specific research questions, studies often only use ‘complete’ teeth by excluding worn specimens or attempting to reconstruct original dimensions. While this approach is often necessary, it inherently overlooks the dynamic morphological transitions that occur as teeth wear. Importantly, each stage of wear represents a distinct morphology, any of which may be subject to selection (Ungar, 2015). Methods such as those employed here, explicitly modelling teeth across multiple wear phases, therefore provide an opportunity to investigate how and why particular features (e.g., individual cusps, aspects of enamel structure, or overall crown form) evolve. Such an approach can complement recent work on the biomechanical and developmental bases of dental formation, eruption, and function (e.g., Cofran & Boughner, 2025; Glowacka & Schwartz, 2021; Monson et al., 2019).

Finally, advances in intraoral scanning technology offer new opportunities for ecological and evolutionary research (see discussion in Towle et al., 2024). These portable, high-resolution tools eliminate the need for moulds or coatings, enabling rapid digital capture of dentitions in the field. Paired with quantitative wear analyses like those used here, intraoral scanning allows for population-level comparisons, integration with microwear and occlusal modelling, and long-term tracking of dental wear in wild animals. In high-wear species such as baboons, we show that these methods remain effective even when occlusal surfaces are heavily worn, because the lateral enamel surfaces provide sufficiently preserved landmarks for reliable alignment. However, over longer intervals (>5 years), progressive buccal and lingual wear will eventually compromise alignment accuracy. Our findings underscore the value of integrating clinical tools with ecological datasets and suggest promising avenues for future research on functional and evolutionary aspects of tooth wear, and in ecological and conservation contexts. Future studies can also split the occlusal surface into regions of particular interest, allowing assessment of changes in particular locations with other types of wear analysis (e.g., microwear) and tissue loss (e.g., cusp attrition in association with fractures).

These methods also hold considerable potential in paleontological research, offering new tools for investigating dental evolution and reconstructing past diets and environments, whether through modern analogues or tooth reconstruction techniques applied to fossil specimens (e.g., Modesto‐Mata et al., 2024). Baboons are especially valuable as comparative models for fossil hominins, owing to their broadly omnivorous diet, mixed terrestrial-arboreal behaviour, and wide geographic range across diverse habitats, many overlapping with those of Plio-Pleistocene hominins. As comparative datasets expand, these influences could be disentangled by examining primates with contrasting ecologies (e.g., leaf-eaters vs frugivores; predominantly arboreal vs largely terrestrial species) and environments, providing deeper insight into the behavioural and environmental drivers of tooth wear.

## Supporting information

10.1017/ehs.2026.10041.sm001 Towle et al. supplementary material 1 Towle et al. supplementary material

10.1017/ehs.2026.10041.sm002 Towle et al. supplementary material 2 Towle et al. supplementary material

## Supplementary material

The supplementary material for this article can be found at https://doi.org/10.1017/ehs.2026.10041

## Research transparency and reproducibility interest

The data that support the findings of this study are openly available in the Supplemental File of this article.
