# Supplementary material for: Primate dental function and evolution: longitudinal 3D tooth wear in wild baboons
Source: Evol Hum Sci. 2026 Mar 24;8:e12. doi: 10.1017/ehs.2026.10041 (PMC13112123; doi:10.1017/ehs.2026.10041)
Supplement: Towle et al. supplementary material 2 — Towle et al. supplementary material [file S2513843X26100413sup002.docx]

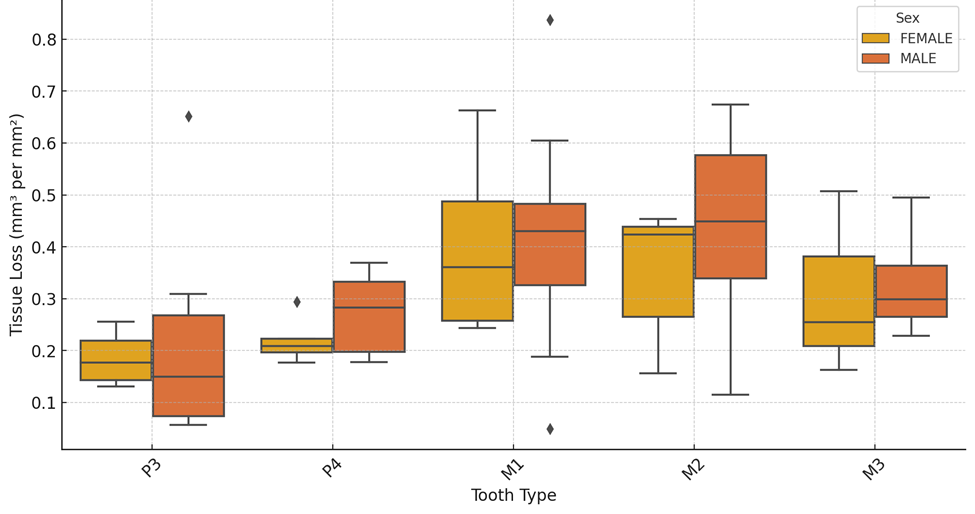


**Supplementary Figure S1.** Box-and-whisker plots showing tissue loss by tooth type, separated by sex.


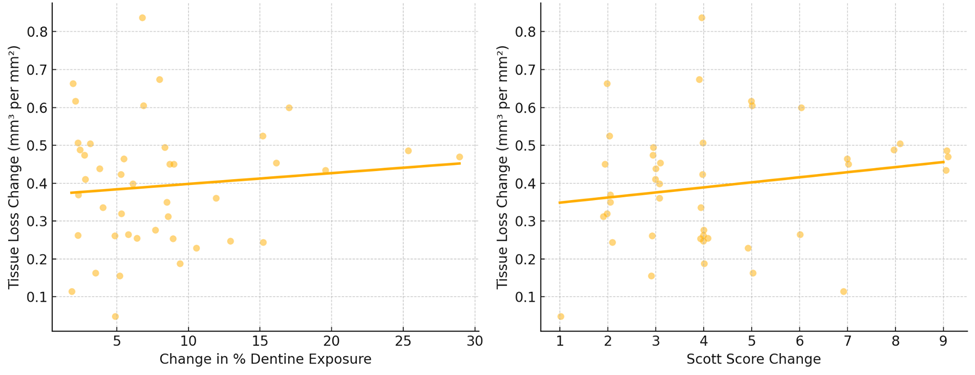


**Supplementary Figure S2.** Scatter plots illustrating the relationships between change in percent of dentine exposure (PDE) and Krueger-Scott score (KSM change, with tissue loss change (mm³ per mm²). Trend lines indicate weak relationships between PDE and tissue loss, and a slightly better correlation between KSM and tissue loss.


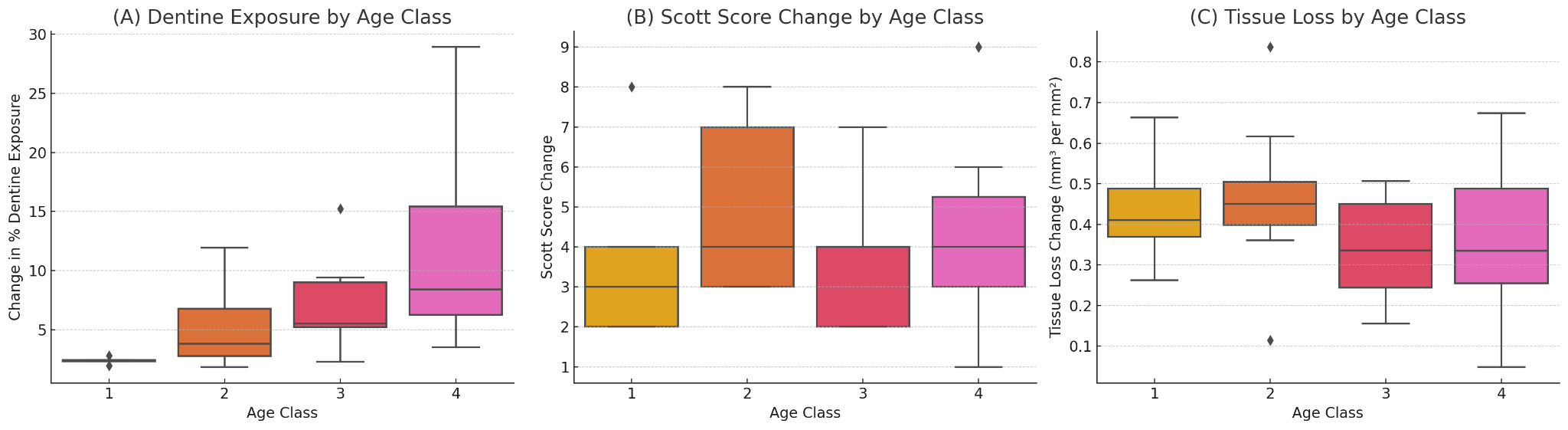


**Supplementary Figure S3.** Boxplots illustrating the distribution of change in percent of dentine exposure (PDE; A), Krueger-Scott score (KSM) change (B), and Tissue loss change (C) across the four age classes. PDE shows a clear increasing trend with age, while KSM change and tissue loss change remain relatively stable.
